# Nanobody and CuS Nanoflower-Au-Based Lateral Flow Immunoassay Strip to Enhance the Detection of Aflatoxin B_1_

**DOI:** 10.3390/foods13121845

**Published:** 2024-06-12

**Authors:** Yiming Zhao, Baoshan He, Danyang Li, Leyan Gao, Wenjie Ren

**Affiliations:** National Engineering Research Center of Wheat and Corn Further Processing, Henan University of Technology, Zhengzhou 450001, China; zyming@163.com (Y.Z.); hebaoshan@126.com (B.H.); 13526992722@163.com (D.L.); cbs_2025@163.com (L.G.)

**Keywords:** CuS nanoflowers-Au, aflatoxin B_1_, nanobody, lateral flow immunoassay, galvanic displacement

## Abstract

In the realm of analysis, the lateral flow immunoassay (LFIA) is frequently utilized due to its capability to be fast and immediate. However, the biggest challenge of the LFIA is its low detection sensitivity and tolerance to matrix interference, making it impossible to enable accurate, qualitative analyses. In this study, we developed a new LFIA with higher affinity and sensitivity, based on a nanobody (G8-DIG) and CuS nanoflowers-Au (CuS NFs-Au), for the detection of aflatoxin B_1_ (AFB_1_) in maize. We synthesized the immunoprobe G8-DIG@CuS NFs-Au, stimulated the in situ development of Au nanoparticles (Au NPs) on Cu NFs by electrical displacement, and obtained Cu NFs-Au for fixing the G8-DIG. G8-DIG@CuS NFs-Au probe-based LFIAs may, in ideal circumstances, use a strip chromatography reader to accomplish sensitive quantitative detection and qualitative visualization. AFB_1_ has a detection range of 2.82–89.56 µg/L and a detection limit of 0.87 µg/L. When compared with an LFIA based on CuS NFs, this sensitivity is increased by 2.76 times. The practical application of this method in corn flour demonstrated a recovery rate of 81.7% to 117%. Therefore, CuS NFs-Au show great potential for detecting analytes.

## 1. Introduction

Mycotoxins are widely present in soil, crops, and animal feed, significantly impacting global agricultural development and causing various physiological and pathological changes in species due to their potent acute toxicity [1,2]. According to estimates from the Food and Agriculture Organization of the United Nations, mycotoxin contamination affects about 25% of agricultural goods and foods derived from them globally, resulting in food and financial losses [3,4,5]. Of these toxins, aflatoxins are the major culprits. A class of secondary metabolites known as aflatoxins is extensively found in nature and is produced by the Aspergillus fungus [1]. One of the most toxic aflatoxins, AFB_1_, is categorized as a Group 1A carcinogen by the International Agency for Research on Cancer (IARC, 1993) due to its teratogenic, mutagenic, and carcinogenic qualities. Therefore, many countries and regions have imposed strict limits on the concentration of AFB_1_ in foodstuffs. For instance, the European Union sets a maximum allowable concentration of 5 μg/kg for AFB_1_ in corn [6], and China and the United States have set the limit at 20 μg/kg [7]. For this reason, it is critical to develop quick, accurate, and affordable ways to detect AFB_1_ in food in order to protect human health and food safety.

At the moment, AFB_1_ is being quantitatively detected using high-performance liquid chromatography (HPLC) [8], thin-layer chromatography (TLC) [9], HPLC-tandem mass spectrometry (HPLC-MS) [10], and other instrumental analytical techniques. These techniques, however, require a lot of time, expensive equipment, and expert operators, making them unsuitable for quick on-site detection. Because of its affordability, ease of use, simplicity, and speed, the LFIA is the most widely used technology for environmental monitoring, clinical diagnosis, and food safety detection. Nevertheless, the major challenge of the LFIA is its low detection sensitivity, which makes it impossible to accurately quantify its analysis in detecting trace amounts of target analytes. Therefore, there is an urgent need to develop strategies to enhance the sensitivity of LFIAs.

Previous studies have shown that optimizing the detection signal is one of the effective ways to improve the detection sensitivity of analytical methods. Due to their superior optical characteristics, gold nanoparticles (Au NPs) are frequently employed as optical nanomaterials in LFIAs [11]. However, they also suffer from the drawback of insufficient sensitivity due to weak signals. In order to enhance the sensitivity and stability of LFIAs, the utilization of signal labels is of the utmost importance. Currently, there exist several prepared signal labels, including dendritic mesoporous silica/gold−silver alloy nanoparticles (dSiO_2_/Au−Ag NP) [12], PDA-coated Hong Kong University of Science and Technology MOFs (HKUST@PDA) [13], Fe_3_O_4_@CuS nanostructures (Fe_3_O_4_@CuS NSs) [14], AuNPs-modified g-C_3_N_4_ nanosheets (Au@CNNS) [15], etc. However, the synthesis of these composite nanoparticles is rather complex. Galvanic displacement is one of the most effective methods in the nanoworld for creating stable composite materials [16,17]. When metal ions with a higher potential come into contact with metals with a lower potential in a solution, electrode replacement occurs [17,18]. By using galvanic replacement, Shu et al. were able to generate Au nanocrystals in situ on CuS nanoplates [19]. This team has also successfully applied galvanic replacement to CuS nanospheres [17]. Bimetallic Ag–Au urchin-like hollow nanospheres (BUHNPs) were created by Zhang et al. through co-reduction and galvanic replacement processes, which allowed for their quick and easy synthesis [20]. Through galvanic displacement, composite nanoparticles were effectively manufactured by merely swirling an aqueous solution containing nanoparticles and AuCl^4−^ ions at ambient conditions, which expedited the synthetic method. CuS nanomaterials are optical and electrical p-type semiconductors with unique characteristics such as a high theoretical capacity and metallic conductivity [16,21]. CuS nanomaterials have been widely applied in optics and electrochemistry [22,23,24]. Currently, CuS nanospheres, CuS nanoplates, and CuS nanoclusters have been utilized in LFIAs, all demonstrating excellent colorimetric performance and increased sensitivity, indicating the superiority of copper sulfide materials [14,17,19].

An antibody is one of the key factors in the preparation of a highly sensitive immunoassay. Compared to traditional monoclonal antibodies, nanobodies have a molecular weight of only 15 kDa, which is one-tenth of the molecular weight of traditional monoclonal antibodies (150 kDa) [25]. Nanobodies also possess strong specificity, high stability, a tolerance to high temperatures, and an ease of fabrication and modification [26]. They thus offer significant potential for practical applications in developing immunosensors. Liao et al. synthesized a nanoantibody and biomimetic mineralized metal–organic framework (MOF)-immune probe for the electrochemical detection of AFB_1_ [27]. Yan et al. used biotinylated nanobody Nb26 and streptavidin-conjugated polymerized horseradish peroxide (SA-PolyHRP) to create a biotin-streptavidin-amplified enzyme-linked immunosorbent test (BA-ELISA) that allows for the sensitive and quick detection of AFB_1_ in cereal [28]. Protein engineering and recombinant technologies have endowed nanobodies with additional properties [29]. Zuo et al. and Li et al. constructed an AFB_1_-specific nanobody Nb26 with enhanced green fluorescent protein (EGFP) [25,29]. Wang et al. achieved targeted binding by synthesizing Nb-Avi [11]. The nanobodies generated exhibit favorable consistency across batches and lend themselves to rapid and large-scale production through straightforward ex vivo expression methods.

In this study, in order to enhance the sensitivity of LFIAs, a new colorimetric label for the test strips was chosen by replacing the traditional signal label, colloidal gold, with CuS NFs-Au. CuS nanoflowers (CuS NFs) provided active sites for the deposition of gold nanoparticles due to their larger specific surface area and greater number of branches. It has been noted that the signals produced by several tiny Au NPs are more potent than the signals produced by one bigger Au NP [30]. Therefore, CuS NFs-Au were prepared by a mild stirring of CuS NFs and HAuCl_4_, allowing gold nanoparticles to grow on the surface of CuS NFs. CuS NFs-Au synthesized by a galvanic displacement reaction typically exhibit high color intensity and enhanced stability. Incorporating nanobodies as a substitute for conventional monoclonal antibodies holds considerable promise. A novel immunosensor based on G8-DIG@CuS NFs-Au has been developed.

## 2. Materials and Methods

### 2.1. Materials

Copper(III) nitrate was purchased from Tianjin Kermel Chemical Reagents Co., Ltd. (Tianjin, China), Thiourea was purchased from Yongda Chemical Reagent Co., Ltd. (Tianjin, China). *N*,*N*-dimethylformamide (DMF) and chloroauric acid (HAuCl_4_) were bought from Shanghai Aladdin Biochemical Technology Co., Ltd. (Shanghai, China). Bovine serum albumin (BSA), ampicillin, bacterial cell lysate, isopropyl-β-D-thiogalactopyranoside (IPTG), and Ni-NTA agarose purification resin were purchased from Sangon Biotechnology Co., Ltd. (Shanghai, China). Polyvinylpyrrolidone (PVP K30, Mw ≈ 58,000) was purchased from Rhawn Reagents (Shanghai, China). Aflatoxin B_1_ (AFB_1_), T-2, ochratoxin A (OTA), deoxynivalenol (DON), and zearalenone (ZEN) were purchased from MEIZHENG BIO-TECH (Rizhao, China). The recombinant vectors pET25b-G8-DIG, *E. coli* BL21, and DIG-BSA were stored in the laboratory.

Ultrapure water (18.25 MΩ) was used for all experiments. Nitrocellulose (NC) membranes of CN 120 were procured from Seebio Biological Technology (Shanghai, China). Absorbent papers, conjugate pads, sample pads, polyvinyl chloride (PVC) substrate plates, a gold-plating film cutting machine, strip cutter, and immunochromatographic quantitative analysis instrument were purchased from Shanghai Jiening Biological Technology Co., Ltd. (Shanghai, China). A desktop high-speed freeze centrifuge was acquired from Hunan Hukang Centrifuge Co., Ltd. (Changsha, China).

### 2.2. Synthesis of CuS NFs

The synthesis of CuS nanoflowers was achieved based on previous reports [31]. A clear blue solution was obtained by dissolving 2 mmol of Cu(NO_3_)_2_·3H_2_O in 20 mL of DMF, followed by stirring for 5 min. Subsequently, 2.0 g of PVP K30 was added to the solution, and stirring continued until complete dissolution. In parallel, 20 mL of DMF was used to dissolve 2 mmol of thiourea, which was then agitated for 5 min. When the stirred copper source solution was combined with the resultant sulfur source solution, the solution turned green instead of blue. Stirring continued for a period of time, and the resulting mixture was transferred into the lined tube of a 100 mL high-pressure reaction kettle. After the reaction kettle was properly sealed and heated to 120 °C for 12 h, it was allowed to naturally cool to ambient temperature. A brown solution was obtained, which was then subjected to three rounds of centrifugation with alternating ultrapure water and anhydrous ethanol. After that, the material was dried in an oven set to 70 °C and refrigerated at 4 °C.

### 2.3. Synthesis of CuS–Au Nanoflowers

The preparation of CuS–Au nanoflowers was carried out following a previously established method with minor modifications [16]. The synthesis of a CuS NFs-Au composite was carried out by dissolving CuS NFs in 100 mL of ultrapure water, followed by their complete dispersion through ultrasonication for 10 min. The mixture was subsequently stirred for a duration of 3 h at ambient temperature following the introduction of 20 µL of HAuCl_4_, without the incorporation of any reducing agents.

### 2.4. Expression of the Nanobody G8-DIG

**Figure 1:**
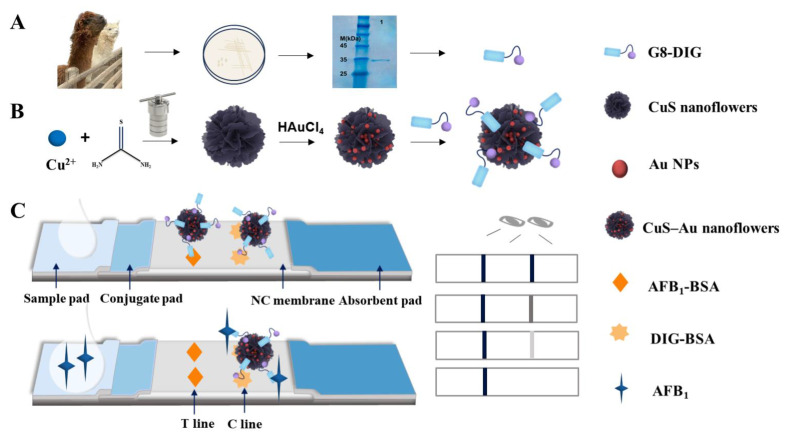
(**A**) Analysis of the G8-DIG using SDS-PAGE. Lane M, pre-stained protein molecular weight marker; lane 1, protein purified by a nickel column. (**B**) The synthesis of CuS NFs, CuS NFs-Au, and G8-DIG@CuS NFs-Au probes, and (**C**) CuS NFs-Au lateral flow immunoassay (LFIA) for AFB_1_ detection.

Glycerol bacteria (Pet25b-G8-DIG) were dipped in an inoculation ring and inoculated on LB solid plates containing ampicillin, using a scribing method, on a clean bench. The plates were incubated upside down overnight in a microbial incubator at 37 °C. A well-grown and suitable single colony was selected from a cultivated agar plate and introduced into a 5 mL of liquid LB medium containing 100 µg/mL of ampicillin. The mixture was then subjected to oscillating incubation at 37 °C and 200 rpm for 12 h in a constant-temperature shaking incubator. The above medium was inoculated into 250 mL of LB liquid medium containing a specific amount of ampicillin and cultured at 37 °C and 220 rpm. When the OD_600_ reached 0.5~0.8, IPTG at a concentration of 0.1 mmol/L was introduced, followed by incubation at 200 rpm, 25 °C, for a duration of 7.5 h. After that, the cells were gathered, and the supernatant was extracted by centrifugation. After lysing the cell pellet with a bacterial lysis buffer, the supernatants were collected by centrifuging the mixture for five minutes at 12,000 rpm and 4 °C. The supernatants were purified using a Ni-NTA His-Tag agarose column. Using sodium dodecyl sulfate–polyacrylamide gel electrophoresis (SDS-PAGE) (Figure 1A), the antibodies’ purity was confirmed. The samples were then dialyzed against phosphate-buffered saline (PBS, 0.1 M, pH 7.4) at 4 °C for a whole day. For later use, the produced G8-DIG was kept in storage at 4 °C. As illustrated in Figure 1A, the experimental results show the presence of the desired protein bands at an approximate molecular weight of 30 kDa, which is in line with their anticipated molecular weight.

### 2.5. Preparation of G8-DIG@CuS-Au (CuS) Nanoflower Probes

The synthesis of G8-DIG@CuS NFs-Au probes is demonstrated in Figure 1A,B as depicted. A total of 900 µL of the CuS NFs-Au (CuS NFs) solution was taken and then adjusted to the desired pH by adding either K_2_CO_3_ or HCl. After adjusting the pH, G8-DIG was added to the mixture and vigorously mixed using a vortex mixer. Next, the solution was incubated for 2 h at 37 °C. To block any leftover active sites, 150 µL of 10% (*w*/*v*) BSA was added, and the mixture was then incubated for a further 30 min. After centrifuging the mixture at 4 °C and 6000 rpm for 10 min, the supernatant was discarded. Finally, the probes were reconstituted using 25 µL of the suspension.

### 2.6. LFIA Preparation

The conjugate pad and sample pad were soaked in a 20 mM borate buffer containing 6.0% seaweed sugar (*w*/*v*), 1.0% BSA (*w*/*v*), and 0.5% Tetron1307 (*w*/*v*) and then dried overnight. Different concentrations of AFB_1_-BSA and DIG-BSA were applied onto an NC membrane at a rate of 0.5 μL/cm using a spray coater to generate a test line (T line) and a control line (C line). Following this, the sample pad, a conjugate pad, and the NC membrane were subsequently affixed onto a PVC backing pad in a sequential manner, as depicted in Figure 1C. There was a 2–3 mm overlap between the conjugate pad and sample pad, and there was also a 2–3 mm overlap between the absorbent paper and the NC membrane. A good capillary force for the probes and sample solution is ensured by this configuration. Finally, the pad was cut into 3 mm wide strips using a strip cutter and stored in a sealed bag for drying.

### 2.7. Principle of the LFIA Based on CuS-Au

The fabrication process and detection principle have been illustrated in Figure 1C. The LFIA is composed of a sample pad, a conjugate pad, an NC membrane, an absorbent paper, and PVC backing. The T line is coated with AFB_1_-BSA, while the C line is coated with DIG-BSA. In the absence of AFB_1_ in the sample, the signal probe is captured by the specific AFB_1_-BSA antigen immobilized on the T line, while the excess is captured by the specific DIG-BSA antigen immobilized on the C line, resulting in the appearance of black lines on both the T and C lines. When AFB_1_ is present in the sample, the signal probe is initially conjugated by AFB_1_ during the mixing process, resulting in a faint or no color intensity on the T line. The color intensity of the T line gradually decreases and ultimately disappears as the concentration of AFB_1_ grows. AFB_1_ can be detected semi-quantitatively with an immunochromatographic quantitative analytical device and qualitatively with the unaided eye.

### 2.8. Key Parameter Optimization

To attain a sufficient level of sensitivity in the identification of AFB_1_, we made numerous experimental parameter adjustments. The pH value of the CuS NFs-Au solution (5.0, 5.5, 6.0, 6.5, 7.0), the amount of G8-DIG (5, 10, 15, 20, 25 μL), the T line concentration (0.1, 0.2, 0.3, 0.4, 0.5 mg/mL), and the volume of probe (1, 2, 3, 4, 5 μL) were optimized. A total of 50 μL of loading buffer (5% PVP K30 + PBST) was added to the sample pad and reacted for 15 min. The color intensity of the T line was measured by the immunochromatographic quantitative analysis instrument. The experiment was carried out three times. At the same time, the positive sample (5% PVP K30 + PBST + 25 µg/L AFB_1_) was added to inhibit the test strip, and the condition with a high inhibition rate was selected as the optimal condition.

### 2.9. Sensitivity Analysis of the CuS NFs-Au LFIA Strip

Different concentrations of an AFB_1_ standard solution were prepared using PBST + 5% PVP K30 sample buffer. The final concentrations of the solution were set at 0.05, 0.1, 0.5, 2.5, 5, 10, 50, 100, 150, 175, and 200 µg/L, and 50 μL of the solution was taken and added to the sample pad. After incubating at 37 °C for 15 min, the T line color intensity (C_T_) was measured using an immunochromatographic quantitative analysis instrument. The logarithm of the AFB_1_ concentration was plotted against the C_T_/C_T0_ ratio to create a color intensity standard curve, where C_T0_ is the color intensity of the negative samples. This standard curve served as the basis for calculating the detection limit. All experiments were conducted three times.

### 2.10. Specificity Experiment for the CuS NFs-Au-LFIA

The specificity of CuS NFs-Au-LFIA was evaluated by detecting AFB_1_ and four other substances: ochratoxin A (OTA), T-2 toxin (T-2), deoxynivalenol (DON), and zearalenone (ZEN). The concentrations of AFB_1_, OTA, T-2, DON, and ZEN tested were set at 150 µg/L. All experiments were conducted in triplicate.

### 2.11. Detection of Actual Samples

An accurate weighing of 5 g of the HPLC-confirmed negative cornmeal was conducted. Subsequently, 1 mL of AFB_1_ standard solution was added separately to each sample, followed by 5 min of agitation and drying at room temperature. Subsequently, 10 mL of extraction reagent (methanol–water, 7:3) was added, and the mixture was subjected to 30 min of oscillation in a shaker at 6000 rpm/min and 4 °C. The supernatant was obtained by collecting it, after centrifugation for 20 min, filtering it through a 0.22 μm filter, and diluting it ten times with a buffer solution to obtain final concentrations of AFB_1_ at 0, 0.1, 1, and 10 µg/L, respectively. Quantitative detection was conducted using the standard curve, and the method’s accuracy was assessed by calculating the recovery rate of the spiked samples. Additionally, the precision of the method was evaluated by calculating the coefficient of variation.

## 3. Results and Discussion

### 3.1. Characterization of CuS and CuS-Au

**Figure 2:**
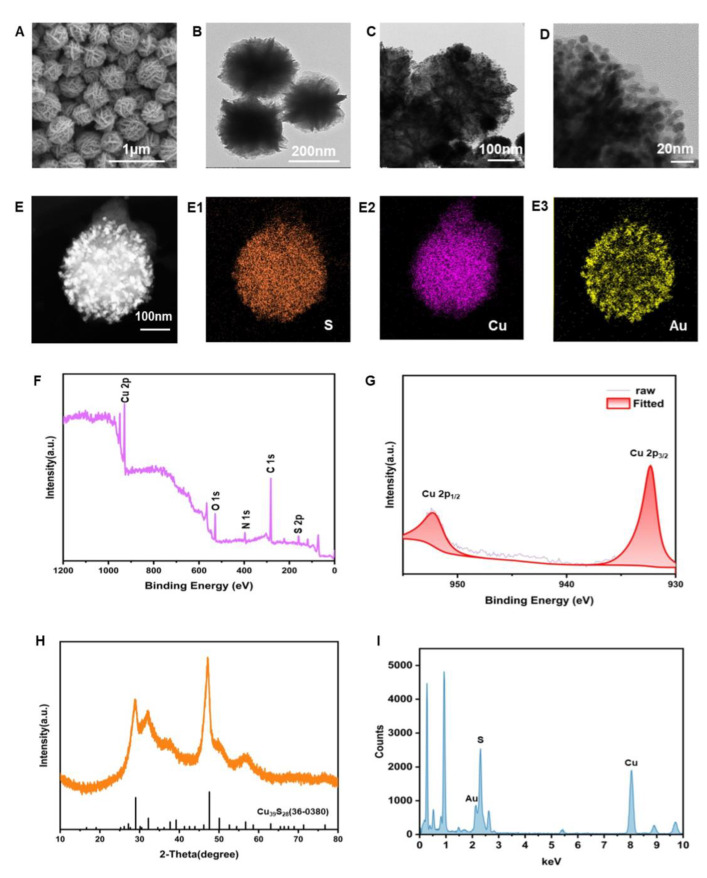
Characterization of prepared CuS NFs, CuS NFs-Au, and G8-DIG@CuS NFs-Au. (**A**) Scanning electron microscope (SEM) image of the CuS NFs. (**B**) Transmission electron microscopy (TEM) image of the CuS NFs. (**C**) TEM image of the CuS NFs-Au. (**D**) High Resolution-TEM (HRTEM) image of the CuS NFs-Au. (**E**) The corresponding element mapping analysis of S, Cu, and Au in the CuS NFs-Au. (**E1**) is the mapping of the S, (**E2**) is the mapping of the Cu, (**E3**) is the mapping of the Au, (**F**) X-ray photoelectron spectroscopy (XPS) survey spectra of CuS. (**G**) XPS survey spectra of the Cu 2p of CuS. (**H**) X-ray diffraction (XRD) pattern of CuS. (**I**) Energy-Dispersive Spectroscopy (EDS) spectrum of CuS NFs-Au.

The morphologies of the nanomaterials created using a scanning electron microscope (SEM) and transmission electron microscopy (TEM) characterization are displayed in Figure 2A,B. With an average diameter of 300 nm, the produced nanomaterials had a high degree of homogeneity, resembled flowers, and were reasonably consistent in size. The TEM images in Figure 2C,D demonstrate the successful deposition of Au NPs onto CuS NFs structures. Through high-resolution transmission electron microscopy (HRTEM) (Figure 2D), it can be seen that particles of a size of about 5 nm are grown on the petals of the synthesized, flower-like material. Through element mapping (Figure 2E,E1–E3), it can be seen that Au elements are distributed on the outside of the material, indicating that the particles on the petals are gold nanoparticles. The element composition and elemental valence state of the CuS NFs were characterized using X-ray photoelectron spectroscopy (XPS), as depicted in Figure 2F, confirming the presence of Cu and S. The Cu 2p spectra of the CuS NFs were examined in order to determine their chemical valence state. This revealed two binding energies at 932.39 eV and 952.29 eV, which correspond to Cu 2p3/2 and Cu 2p1/2, respectively (Figure 2G). The X-ray diffraction (XRD) pattern of the CuS NFs exhibited diffraction peaks at 28.86°, 32.13°, 47.08°, 49.74°, and 56.32° corresponding to the hexagonal phase of Cu39S28 (JCPD file No. 36-0380) [31], thus confirming the synthesis of CuS NFs (Figure 2H). The EDS mapping indicated that the synthesized material predominantly consisted of three elements: Cu, S, and Au. This further validated the successful in situ growth of Au nanoparticles on CuS NFs (Figure 2I).

### 3.2. Optimization of the Key Parameters for an LFIA Strip Based on CuS NFs-Au

**Figure 3:**
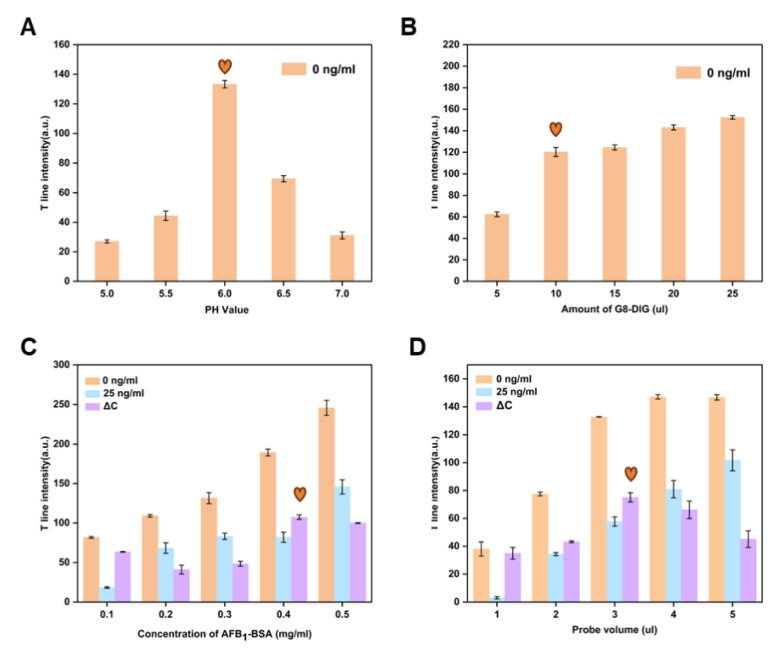
Optimization of CuS NFs-Au LFIA strip. Effects of (**A**) pH, (**B**) amount of G8-DIG, (**C**) AFB_1_-BSA concentration, and (**D**) volume of probe. (The heart represents the optimal condition).

pH is a key factor that influences the stability of the binding between nanobodies and materials. It is evident from Figure 3A that the T line’s color intensity changes most noticeably at a pH of 6.0. For the conjugation of G8-DIG with CuS NFs-Au, a pH of 6.0 was therefore ideal.

To optimize the G8-DIG nanobody, a gradient optimization approach was employed. Under the optimal pH conditions, 5, 10, 15, 20, and 25 μL of G8-DIG were added to five centrifuge tubes. The color intensity of the T line was observed visually. We decided to use 10 μL of G8-DIG since there was no significant change in the color intensity of the T line when 10 μL or 15 μL of G8-DIG were added. This choice was made, based on the findings in Figure 3B, to ensure the preservation of antibodies while maintaining the desired level of coloration in the T line.

The concentration of AFB_1_-BSA on the T lines was optimized according to ΔC = C_T0_ − C_T1_, where C_T0_ represents the color intensity of the T lines of negative samples and C_T1_ represents the color intensity of T lines with 25 µg/L of AFB_1_ samples added. ΔC is an indicator of the competition inhibition ratio and the LFIA’s sensitivity. Consequently, the parameters that resulted in the highest ΔC were selected as optimal. According to Figure 3C, as the concentration of the T line increases, the color intensity also gradually increases. When the concentration of the T line is 0.4 mg/mL, the ΔC is maximized, indicating the highest inhibition rate under this condition. Therefore, the ideal concentration of AFB_1_-BSA on the T line was determined to be 0.4 mg/mL. Additionally, the effect of probe volume on ΔG was examined. According to Figure 3D, when the probe volume is 3 μL, the ΔC is maximized, making 3 μL the optimal probe volume.

### 3.3. Comparison of Sensitivity of the CuS-Au-LFIA and CuS-LFIA

**Figure 4:**
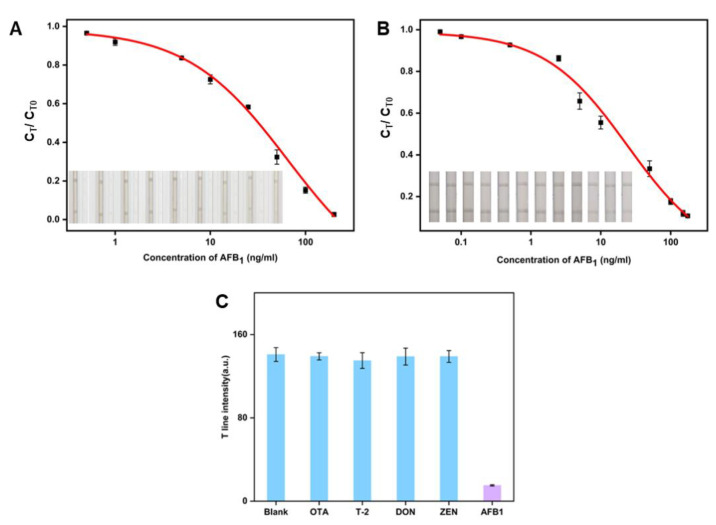
Assay performances of the LFIAs for AFB_1_ detection. (**A**) AFB_1_ detection by the CuS NFs-LFIA and visual pictures (*n* = 3). (**B**) AFB_1_ detection by the CuS NFs-Au-LFIA and visual pictures (*n* = 3). (**C**) The specific results of AFB_1_ and other toxins detected by the CuS NFs-Au-LFIA.

The detection of AFB_1_ in the LFIA test strips was performed using CuS NFs-Au and CuS NFs. The G8-DIG@CuS NFs-Au and G8-DIG@CuS NFs, two different kinds of immunological probes, were prepared in advance. As the concentration of AFB_1_ increased in the qualitative detection trials (Figure 4A,B), the contrast (C_T_) progressively decreased. The logarithmic value of the AFB_1_ concentration was used as the abscissa (X) and C_T_/C_T0_ as the ordinate (Y) to create a standard curve for the CuS NFs-Au-LFIA and CuS NFs LFIA. Using the logistic equation y = A2 + (A1 − A2)/[1 + (x/x0^p^)], the standard curve was computed. The CuS NFs-Au-LFIA’s IC_10_ was determined to be 0.87 µg/L, with a good linear range of 2.82–89.56 µg/L (Figure 4B). The IC_10_ of the CuS NFs-Au-LFIA was 2.76 times lower than that of the CuS nanoflowers LFIA (IC_10_ = 2.4 µg/L L).

### 3.4. Specificity Experiment

Figure 4C displays the results of a specific experiment involving OTA, T-2, DON, and ZEN. At a concentration of 150 µg/L of AFB_1_, a notable diminishment in the visibility of the gray band on the T line is noted, contrasting with the continued presence of the gray band on the T line with the same concentration levels of other specified substances. These findings indicate that the established CuS NFs-Au-LFIA exhibits a favorable specificity for the detection of AFB_1_.

### 3.5. Practical Application in Actual Samples

Using its recovery rate and coefficient of variation (CV), the CuS NFs-Au-LFIA method was used to assess the accuracy of AFB_1_ detection. The recoveries of AFB_1_ in corn flour ranged from 81.7% to 117%, and the CV was less than 11.5% (Table 1). These results demonstrate the excellent accuracy of the CuS NFs-Au LFIA method. To further verify the feasibility and accuracy of CuS NFs-Au-LFIA, standardized corn samples containing AFB_1_ at a known concentration of 0.535 µg/L were procured from the Academy of Sciences of the State Food and Material Reserve Bureau. The concentration of AFB_1_ in corn flour was determined to be 0.58 µg/L when using the CuS NFs-Au-LFIA method, with a recovery rate of 108%.

## 4. Conclusions

In conclusion, we have developed an LFIA for the visual and quantitative detection of AFB_1_, utilizing CuS NFs-Au as signal labels. The preparation of CuS NFs with the in situ growth of gold nanoparticles through galvanic displacement eliminates the need for a reducing agent, simplifying the process. Furthermore, the combination of CuS NFs-Au (CuS NFs) with G8-DIG through electrostatic adsorption avoids the need for complex chemical conjugation. This method demonstrates a detection limit of 0.87 µg/L for AFB_1_, with a detection range of 2.82-89.56 µg/L. Compared to the CuS NFs-LFIA, the sensitivity of this assay has been improved by 2.76 times. Moreover, this assay shows no cross-reactivity with other mycotoxins and achieves a practical sample recovery rate of 81.7%-117%. The CuS NFs-Au-LFIA may offer a new option for the detection of other substances.

## Figures and Tables

**Table 1:** Recovery and CV results for corn flour samples.

Sample	Spiked (µg/L)	Detected (µg/L)	Recovery (%)	CV (%)
Corn flour	0.1	0.09 ± 0.01	90	11.1
	1	1.17 ± 0.12	117	10.3
	10	8.17 ± 0.16	81.7	1.9

## Data Availability

The original contributions presented in the study are included in the article, further inquiries can be directed to the corresponding author.
